# Beyond Mean Scores: Sex Differences in Literacy, Numeracy, and Problem-Solving as Intraindividual Strengths Across Age Groups

**DOI:** 10.3390/jintelligence14010012

**Published:** 2026-01-06

**Authors:** Marco Balducci, Waseem Haider

**Affiliations:** 1 Department of Psychology and Speech Language Pathology, INVEST Flagship Research Center, University of Turku, 20014 Turku, Finland; 2 Department of Social Research, INVEST Flagship Research Center, University of Turku, 20014 Turku, Finland; waseem.w.haider@utu.fi

**Keywords:** literacy, numeracy, problem-solving, sex differences, STEM fields, intraindividual strengths

## Abstract

The underrepresentation of women in science, technology, engineering, and mathematics (STEM) fields has been a longstanding issue. Traditionally, research on sex differences in cognitive abilities has focused on mean scores, which are often trivial and do not appear to explain sex disparities in STEM participation. Recently, intraindividual strengths have been proposed as a more relevant factor; they reflect an individual’s relative advantage in one skill (e.g., literacy) compared with a set of related skills (literacy, numeracy, and problem-solving). Previous studies have primarily examined younger cohorts, and intraindividual strengths remain unexplored across the lifespan. In this study, we employed data from the second cycle of the Programme for the International Assessment of Adult Competencies (PIAAC) including 157,525 individuals from 30 countries to assess sex differences in literacy, numeracy, and problem-solving as intraindividual strengths across five age groups (16–24, 25–34, 35–44, 45–54, and 55+ years). Consistent with previous research, women outperformed men in literacy, while men outperformed women in numeracy. These patterns were observed universally across countries and age groups. In contrast, no sex differences were observed in problem-solving. Future research should move beyond mean scores to focus on intraindividual strengths, as they may be more relevant for understanding sex disparities in STEM.

## 1. Introduction

Despite numerous efforts to balance participation, women remain substantially underrepresented in many inorganic science, technology, engineering, and mathematics (STEM) fields, such as physics and computer science (Kahn & Ginther, 2017). The reasons for this underrepresentation are at the core of a long-lasting debate, as sex disparities in STEM participation are believed to contribute to broader inequalities, including the gender pay gap (Cech, 2013). Some scholars have proposed that these disparities emerge from the roles imposed on men and women (Eagly & Wood, 2012), while others have argued that sex differences in cognitive abilities (Wai et al., 2010a), along with several other factors (e.g., preferences; Su et al., 2009), also play a role. Among cognitive abilities, occupation-related skills—such as verbal and numerical skills—have been of particular interest because of their links to career choices and retention (Kahn & Ginther, 2017). Traditionally, research has focused on mean scores when assessing these skills, which reveal sex differences that are rather small and fail to capture strong correlations with whether individuals choose or opt out of STEM fields (Halpern & Wai, 2019).

A new line of research has proposed moving beyond mean scores and focusing on intraindividual strengths, as they can provide a more nuanced perspective on the underrepresentation of women in STEM (Balducci, 2023). Intraindividual strengths refer to an individual’s comparative advantage in one skill within a set of related skills, independent of their overall achievement (i.e., mean scores across all considered skills) and how they fare compared with their peers (Stoet & Geary, 2018). In other words, these strengths measure an individual’s relative performance within their own skill set, regardless of that of others. For example, someone who scores higher in numeracy than in literacy and problem-solving, respectively, would have their primary intraindividual strength in numeracy, followed by literacy, and lastly, problem-solving.

The argument is derived from expectancy-value theory (Eccles, 1983), which suggests that intraindividual strengths play a key role in shaping career decision-making processes. Individuals with strengths in numerical domains, such as mathematics, are more likely to pursue careers in STEM, as they expect to succeed in these fields. Conversely, those with strengths in verbal domains, such as reading, are more likely to choose interpersonal-oriented occupations (Bernstein et al., 2019). Indeed, these patterns have been described in numerous studies, from those focused on mathematical precocious youth to large-scale analyses conducted in Sweden and the U.S. (Dekhtyar et al., 2018; Wai et al., 2010b; Webb et al., 2002).

The most relevant pattern related to sex disparities in STEM participation indicates that boys often have intraindividual strengths in numerical domains (e.g., mathematics), whereas girls often have strengths in verbal domains (e.g., reading) (Stoet & Geary, 2018). These differences have been reliably observed across countries and over time, and they are significantly larger than those seen in mean scores (Balducci et al., 2024).

### 1.1. Intraindividual Strengths vs. Ability Tilt

Intraindividual strengths are not the only type of relative advantage studied in the literature; the concept is closely related to ability tilts (Wai et al., 2018). However, the two differ significantly in both their theoretical and computational basis. Ability tilts capture a student’s relative advantage in one domain (typically numerical) compared to another (typically verbal) through an algebraic subtraction (e.g., mathematics minus reading scores). This pairwise calculation presents two limitations. First, if the student’s best subject falls outside the two domains considered, it will be overlooked. Second, and more crucially, the calculation neglects overall performance. Hence, ability tilts may not fully capture the relative advantage described by expectancy-value theory, which emphasizes general competence. Despite these limitations, sex differences in ability tilts have been largely documented, with the pattern resembling that found in intraindividual strengths: girls generally tilting towards verbal domains and boys tilting towards numerical domains (Coyle, 2025; Wai et al., 2010a, 2018).

Compared to ability tilts, intraindividual strengths offer a broader, more comprehensive measure by quantifying the deviation of each domain from a student’s overall ability score. In fact, unlike ability tilts, intraindividual strengths incorporate the student’s mean performance across all domains (e.g., the average of mathematics, reading, and science in a test like PISA) as a baseline or expected achievement. Mathematically, they are calculated as the difference between the specific domain score and the mean across all subjects (e.g., Intraindividual reading strengths = reading score minus overall performance). Consequently, intraindividual strengths reflect ability tilt while simultaneously accounting for overall performance, suggesting they better align with expectancy-value theory. Another key advantage is the scalability of intraindividual strengths, as the concept can easily be extended beyond core subjects to include additional academic skills (like critical thinking). This allows for the model to account for mean scores across all considered subjects, offering a more flexible understanding of individual abilities than the strict pairwise comparisons of ability tilts.

### 1.2. Current Study

Research on sex differences in intraindividual strengths has generally focused on high school students, and it remains unclear whether the sex differences described in adolescents (e.g., Balducci et al., 2025) persist and are consistent across age groups. In fact, men and women exhibit different age-related cognitive decline trajectories, with women, at first, demonstrating greater resilience, particularly in verbal skills (McCarrey et al., 2016). However, this resilience tends to fade with older age, often leading to a reduction in sex differences (Dong et al., 2025). It is possible that the initial disparity in cognitive decline could exacerbate pre-existing sex differences in intraindividual strengths during mid-adulthood when men and women are still actively establishing their careers. The potential impact could be particularly pronounced for women’s retention in STEM fields.

In this preregistered study, we employ data from a recently released wave (i.e., December 2024) of the Programme for the International Assessment of Adult Competencies (PIAAC), which included 157,525 individuals across 30 countries, to analyze sex differences in intraindividual strengths in numeracy, literacy, and problem-solving skills across five age groups (16–24, 25–34, 35–44, 45–54, and 55+ years).

Based on the previous literature on sex differences in intraindividual strengths and age-related cognitive decline, we formulated the following hypotheses:

1. Sex differences in intraindividual strengths are expected to favor men in numeracy skills and women in literacy, both overall and across age groups.
2. These differences are expected to initially widen in early age groups due to women’s heightened resilience to cognitive decline, before starting to converge in later stages of life.
3. In contrast, we do not expect to find significant differences in problem-solving overall and across age groups, as this skill relates to the ability to achieve a goal in a dynamic situation (OECD, 2021) and likely relies on cognitive processes related to both numeracy and literacy.

Of note, throughout the manuscript, we refer to sex rather than gender. In the PIAAC, participants were asked to report their gender but were only given a binary option: male or female. This binary classification likely aimed to capture the biological aspect of sex and also does not allow for a full analysis of gender differences in intraindividual strengths, as gender requires a multidimensional rather than binary framework.

## 2. Materials and Methods

The study’s secondary data analysis preregistration can be found at: https://osf.io/58r4f.

### 2.1. Sample

We examined sex differences in intraindividual strengths across age groups using data from the second cycle of the PIAAC. This cycle comprises 30 countries and 157,525 participants ranging from 16 to 65 years of age. The first PIAAC cycle was not considered because its data collection spanned six years, potentially introducing confounding factors related to variations in the timing of data collection. Additionally, participants had the option to complete the reading (part of the literacy test) and problem-solving assessments in the first cycle, whereas these components became mandatory in the second cycle.

We excluded individuals with missing data on sex (*N =* 300) or age (*N* = 336), resulting in a final sample of 157,189 participants across 30 countries.

Note that the second PIAAC cycle surveyed 31 countries, though data for the Netherlands were not yet available. Furthermore, data for Belgium and the U.K. are limited to the Flemish region and England, respectively.

### 2.2. Measurements

#### 2.2.1. PIAAC

The PIAAC was developed by the Organisation for Economic Co-operation and Development (OECD) and measures the cognitive as well as workplace skills required to engage in everyday adult life. Data collection for the second cycle took place between 2022 and 2023, surveying a representative sample of individuals. The assessment covered three main domains: literacy, numeracy, and problem-solving skills in technology-rich environments, with questions often framed as real-world problems. Literacy refers to the ability to engage with written language. It focuses on text interpretation and comprehension rather than writing skills; that is, it assesses the ability to read and understand rather than the ability to write. Numeracy refers to the capacity to interpret and employ mathematical information, whereas problem-solving focuses on the ability to use digital tools to perform practical tasks (OECD, 2021). Each skill is measured using a set of 10 plausible values (PVs), which are generated from the posterior distribution of observed item responses to reflect uncertainty about an individual’s true proficiency level. These values range from 0 to 500 and are scaled using a combination of item response theory and a latent regression model. The PIAAC is administered electronically; however, a paper-based version was provided to individuals without access to a computer. Notably, the reading component of literacy skills was assessed only with a paper-and-pencil test, whereas problem-solving was assessed exclusively in a computer-based format (OECD, 2019).

#### 2.2.2. Relation Between the PIAAC and PISA

Previous research on intraindividual strengths has primarily used data from the PISA (Balducci et al., 2024; Stoet & Geary, 2018), with some exceptions (Dekhtyar et al., 2018). The PISA, conducted every three years, assesses high school students’ (aged 15 years and 3 months to 16 years and 2 months) skills in mathematics, reading comprehension, and science literacy.

The PIAAC and PISA are both administered by the OECD and share conceptual similarities. They aim to measure skills and their application in everyday, real-life situations, rather than simply assessing raw competencies. Additionally, the PIAAC and PISA construct their skill measures based on a common framework that includes three key components: content (i.e., knowledge), cognitive processes, and context (i.e., the different situations in which knowledge is applied). Specifically, literacy in the PIAAC closely aligns with reading in the PISA in terms of the cognitive processes being assessed. Similarly, numeracy in the PIAAC and mathematics in the PISA share a common conceptual foundation (OECD, 2024). The exception is problem-solving in the PIAAC, which has no counterpart in the PISA. Finally, while the PIAAC and PISA were not psychometrically designed for direct comparison, recent studies have reported concordance between the two assessments (Borgonovi et al., 2017; Pokropek & Borgonovi, 2020).

Regarding the cohorts in both assessments, the PISA’s target population consists of high school students aged 15 years and 3 months to 16 years and 2 months. In contrast, the PIAAC has a broader target population, including individuals aged 16–65 years. Consequently, several PISA cohorts are also participating in the second PIAAC cycle. Specifically, these cohorts took the PISA test between 2000 and 2015 in the countries that were involved in both assessments (OECD, 2024).

### 2.3. Analysis

We conducted all analyses using STATA 18 and set an alpha level of 0.05. Graphs were generated using R 4.4.3. Additionally, we followed the recommendations for analyzing data with PVs provided by the PIAAC. Each analysis was performed separately for each PV, and the results were averaged over (Maehler & Rammstedt, 2020).

#### 2.3.1. Age Groups

Yearly age data are available for only 14 countries; however, the PIAAC also provides age group classifications for all individuals. We used this categorization to divide our sample into five age groups: 16–24, 25–34, 35–44, 45–54, and 55+ years. Based on the existing literature regarding age-related cognitive decline and corresponding sex differences (McCarrey et al., 2016), on average, a one-year increase in age is associated with a 0.02–0.03 SD decline in cognitive abilities. This association appears to remain consistent in magnitude as individuals age, which, in the categorization above, corresponds to a decline of approximately 0.2–0.3 SDs in cognitive abilities every 10 years (Salthouse, 2012).

#### 2.3.2. Intraindividual Strengths Computation

We computed intraindividual strengths following the procedure detailed by Stoet and Geary (2018) and Balducci et al. (2024), summarized below.

1. Initially, we computed each participant’s overall PIAAC performance by averaging their PVs from each domain, resulting in a variable labeled PV-Gen. Specifically, PV1-GEN is calculated as (PV1-Literacy + PV1-Numeracy + PV1-ProblemSolving)/3. This procedure was repeated for all 10 PVs. The resulting PVs-Gen were then standardized (mean 0, SD 1) on a country-by-country basis to obtain the Z-Gen scores.
2. Each PV from the individual domains (literacy, numeracy, and problem-solving) was also standardized separately within countries, generating the *Z*-Literacy, *Z*-Numeracy, and *Z*-ProblemSolving scores.
3. To calculate individuals’ intraindividual strengths, we subtracted the *Z*-Gen scores from the domain-specific *Z* scores. For example, the intraindividual strength in literacy skills was calculated as *Z*1-Literacy minus *Z*1-Gen, where “1” represents the first PV. This procedure was repeated separately for each domain and PV.
4. Finally, we calculated the average intraindividual performance of men and women at the country level and subtracted them from one another to determine sex differences. Subtraction was performed both overall and separately across age groups. Note that sex differences in intraindividual strengths can also be computed using the *repest* command (see below) provided by the PISA, running regression models with “sex” as the main predictor after computing the intraindividual strengths in step 3. These models reveal sex differences identical to those calculated by subtraction.

#### 2.3.3. Analytical Strategy

The analytical strategy comprised three steps:

1. First, we assessed sex differences in mean scores—rather than intraindividual strengths—in literacy, numeracy, and problem-solving overall, by country, and across age groups. This approach enabled a direct comparison between the magnitude of sex differences in mean scores and intraindividual strengths. We used the *repest* command in STATA to calculate these differences at the country level. This command employs BRR replicate weights, ensuring that data clustering and sampling errors are properly accounted for, resulting in unbiased standard errors.
2. Next, we examined the sex differences in intraindividual strengths across each domain and compared them with the differences observed in the overall scores. This analysis was conducted for the entire sample, by country, and across age groups.
3. To further investigate the relationship between sex differences in intraindividual strengths and age, we extended our analysis using a linear regression model (Ordinary Least Squares) with a sex-by-age interaction and a bootstrap method with 1000 iterations, as follows:
  $${IIS}_{ic}=\beta 0+\beta_{1}{Sex}_{c}+\beta_{2}{Age}_{c}+\beta_{3}{\left(Sex\times Age\right)}_{c}+\in_{c}$$
  where ${IIS}_{ic}$ represents the mean intraindividual strength in a given domain for men and women in country *c*.

## 3. Results

### 3.1. Sex Differences in Mean Scores

We first analyzed sex differences in the mean scores of the entire sample, across countries and age groups. By initially focusing on mean scores, we were able to compare the magnitude and patterns of these sex differences with those observed for intraindividual strengths.

#### 3.1.1. Full Sample

Examining sex differences in mean scores across the full sample of 157,189 participants, the results indicated no significant advantage for men or women in literacy skills (*p* > .05). However, men outperformed women in both numeracy and problem-solving, although the effect sizes were small for numeracy (standard deviations (SDs) 0.17, 95% confidence interval (CI) = [0.20, 0.11], *p* < .001) and negligible for problem-solving (SDs 0.05, 95% CI = [0.08, 0.02], *p* = .005).

**Figure 1:**
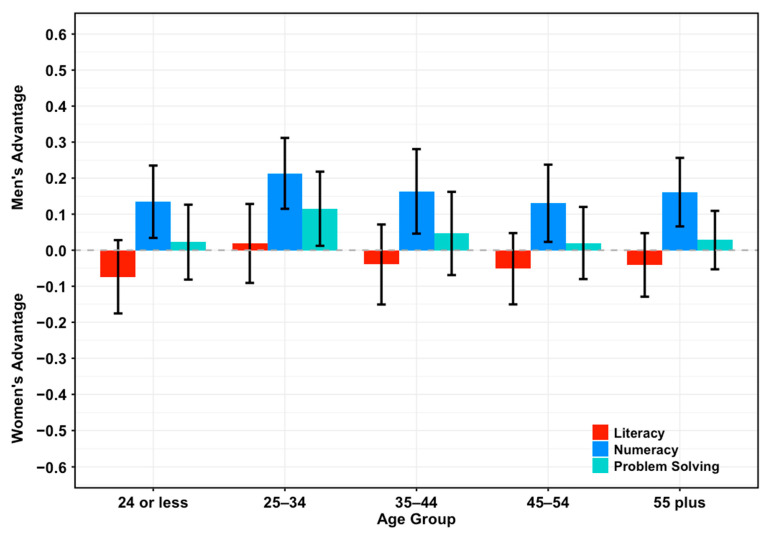
**Sex Differences in mean scores across age groups.** Standardized sex differences (95% CI) in mean literacy (red), numeracy (blue), and problem-solving (cyan) across age groups for the entire sample. Negative values indicate an advantage for women, whereas positive values indicate an advantage for men.

Dividing the entire sample by age group, we found results similar to those described above for literacy and numeracy, while problem-solving showed a slightly different pattern (Figure 1). Sex differences in literacy approached zero and were not significant across age groups. In contrast, numeracy revealed a small but consistent advantage for men, with coefficients ranging from 0.21 SDs (95% CI = [0.31, 0.12], *p* < .001) for the 25–34 years group to 0.13 SDs (95% CI = [0.24, 0.02], *p* = .02) for the 45–54 years group. Ordinary least squares (OLS) models with a sex-by-age interaction revealed no variation in the magnitude of this advantage across the lifespan. Lastly, for problem-solving, there were no sex differences in four out of five age groups (*p* > .05), with only the 25–34 years group showing a trivial difference favoring men, of 0.12 SDs (95% CI = [0.01, 0.22], *p* = .03).

#### 3.1.2. Across Countries

Turning to sex differences in mean PIAAC scores across the three domains by country, we observed substantial variability. In most countries, neither men nor women demonstrated an advantage in literacy skills. However, in a few countries—Croatia, Estonia, Finland, France, Germany, Hungary, Israel, Latvia, Lithuania, New Zealand, Norway, Poland, and Singapore—sex differences favored women, but these differences were usually small (see the Supplementary Material for details). The largest sex differences were observed in New Zealand (−0.20 SDs, 95% CI = [−0.33, −0.07], *p* = .002) and the smallest in France (−0.06 SDs, 95% CI = [−0.12, −0.00], *p* = 0.04).

For numeracy, men generally outperformed women in most, but not all, countries. No differences were observed in Croatia, New Zealand, Poland, or the Slovak Republic (*p* > .05). Among the significant results, the coefficients ranged from 0.31 SDs (95% CI = [0.23, 0.38], *p* < .001) in Switzerland to 0.08 SDs (95% CI = [0.01, 0.15], *p* = 0.02) in Hungary.

Lastly, the results for problem-solving skills mirrored the pattern observed for literacy. Overall, sex differences were negligible (*p* > .05), with a few countries showing a slightly more significant advantage for men; these countries included Austria, Belgium, Chile, Korea, Lithuania, Portugal, Singapore, Switzerland, and the United Kingdom. Portugal had the largest sex differences (0.15 SDs, 95% CI = [0.07, 0.23], *p* < 0.001), whereas Belgium had the smallest (0.07 SDs, 95% CI = [0.01, 0.14], *p* = 0.03).

Across all age groups, there was again considerable variability in sex differences. In most countries, there were no notable differences in literacy between men and women, regardless of age. However, a few countries displayed an advantage for women, but only within specific age groups (see the Supplementary Material for further information). Estonia was an exception, with women consistently outperforming men in literacy across all age groups. Notably, Estonia also showed the largest sex differences in literacy, favoring women, where those aged 24 years or younger outperformed men by −0.28 SDs (95% CI = [−0.38, −0.17], *p* < .001). Meanwhile, Canada and Singapore were the only countries where men had an advantage in literacy. This advantage was limited to the 35–44 years age group, with Singapore displaying the largest sex differences (0.16 SDs, 95% CI = [0.038, 0.28], *p* = .01).

In terms of numeracy, men outperformed women in most countries and age groups, although we found several exceptions to this trend (see the Supplementary Material for details). The largest sex difference, 0.42 SDs (95% CI = [0.28, 0.56], *p* = .001), was observed in Canada among individuals aged 35–44 years. In contrast, the smallest difference (0.11 SDs, 95% CI = [0.01, 0.22], *p* = .03) was found in France among those aged 24 years or younger.

We found significant sex differences in problem-solving in very few instances. These differences typically approached zero across all age groups and countries. Statistically significant differences generally favored men, although the effect sizes were consistently small. For example, the largest difference was 0.23 SDs (95% CI = [0.58, 0.40], *p* = .009) in the U.S. among participants aged 25–34 years. One exception was Estonia, where women outperformed men by −0.12 SDs (95% CI = [−0.24, −0.01], *p* = .04) in problem-solving among those aged 24 years or younger.

Overall, across countries and age groups, we found substantial variation, with little to no evidence of sex differences in literacy or problem-solving skills. However, in numeracy, the differences tended to favor men, but these were generally small in magnitude.

### 3.2. Intraindividual Strengths

#### 3.2.1. Full Sample

When examining sex differences in intraindividual strengths for the entire sample (*N* = 157,189), we observed a distinct pattern compared with the mean scores. In line with our hypotheses, women outperformed men in literacy as an intraindividual strength by approximately −0.39 SDs (95% CI = [−0.43, −0.34], *p* < .001), while men outperformed women in numeracy by a similar degree (0.37 SDs, 95% CI = [0.32, 0.41], *p* < .001). Notably, the magnitude of these advantages was considerable, unlike the small or non-significant sex differences observed in the mean scores. In contrast, problem-solving showed only marginally significant and negligible sex differences favoring women (−0.05 SDs, 95% CI = [−0.09, 0.00], *p* = .05).

**Figure 2:**
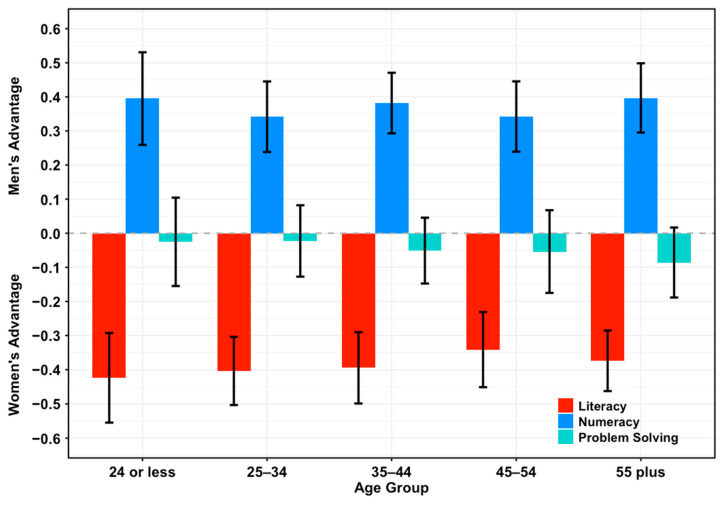
**Sex differences in intraindividual strengths across age groups.** Standardized sex differences (95% CI) in literacy (red), numeracy (blue), and problem-solving (cyan) as intraindividual strengths across age groups for the entire sample. Negative values indicate an advantage for women, whereas positive values indicate an advantage for men.

The same pattern was also noted across age groups (Figure 2). Sex differences in literacy as an intraindividual strength were consistently significant and substantially favored women, regardless of the age group analyzed. Coefficients ranged from −0.42 SDs (95% CI = [−0.55, −0.29], *p* < .001) for the youngest group to −0.34 SDs (95% CI = [−0.45, −0.23], *p* < .001) for the 45–54 years group. For numeracy, men showed a non-trivial advantage in intraindividual strength over women across age groups, with the largest coefficient (0.40 SDs (95% CI = [0.30, 0.50], *p* < .001) observed for the 55+ years group and the smallest (0.34 SDs, 95% CI = [0.24, 0.45], *p* < .001) for the 45–54 years group. Problem-solving showed a trend with sex differences in intraindividual strength favoring neither men nor women in each age group.

OLS models, including a sex-by-age interaction, revealed no significant changes in the magnitude of sex differences in literacy and numeracy as intraindividual strengths across the lifespan, providing no support for our third hypothesis.

#### 3.2.2. Across Countries

Sex differences in literacy as an intraindividual strength were universal, favoring women in all 30 countries analyzed. In other words, there was no country in which men had an advantage over women in literacy as an intraindividual strength. These differences were not statistically significant only in the Slovak Republic. We found that the largest sex differences were in Estonia (−0.61 SDs, 95% CI = [−0.69, −0.53], *p* < .001), while the smallest differences were in Poland (−0.10 SDs, 95% CI = [−0.19, −0.005], *p* = 0.04).

Sex differences in numeracy exhibited the same remarkable consistency as those in literacy, but here, the advantage was in favor of men. In all countries, men had significantly higher intraindividual strengths in numeracy, with none of the countries showing the opposite trend. Poland and the Slovak Republic were exceptions, where men’s advantage was not significant (*p* > .05). These differences were particularly pronounced in Estonia (0.56 SDs, 95% CI = [0.48, 0.63], *p* < .001) and Canada (0.56 SDs, 95% CI = [0.42, 0.68], *p* < .001), whereas the smallest differences were observed in Singapore (0.14 SDs, 95% CI = [0.02, 0.24], *p* = 0.02).

Again, problem-solving deviated from the general pattern, showing no significant differences in intraindividual strength. However, there were exceptions in Canada, Japan, Spain, Sweden, Switzerland, and the United Kingdom, where women’s advantage emerged. Among these countries, Canada showed the largest—although still trivial—sex differences in problem-solving as an intraindividual strength (−0.21 SDs, 95% CI = [−0.29, −0.13], *p* < .001).

Next, we examined the correlations (Spearman’s ρ) between sex differences in intraindividual strengths in literacy and numeracy at the country level. Notably, we found a very strong positive association between the two (ρ = 0.90, 95% CI = [0.81, 1.00], *p* < .001). In other words, the greater the female advantage in literacy as an intraindividual strength, the greater the male advantage in numeracy. The OLS models showed that a 1 SD increase in sex differences in literacy as an intraindividual strength was associated with a 0.88 SD increase in sex differences in numeracy (95% CI = [0.71, 1.06], *p* < .001).

**Figure 3:**
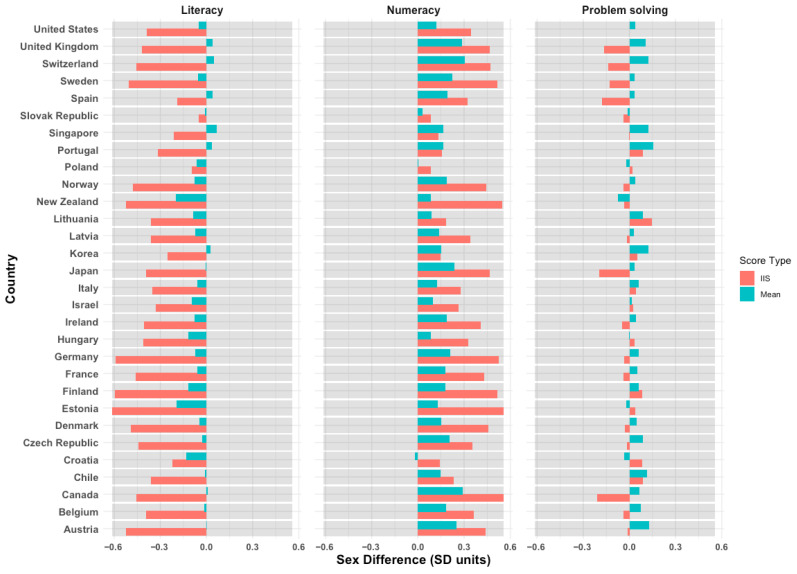
**Sex differences in mean scores and intraindividual strengths across countries.** Standardized sex differences in mean scores (cyan) and intraindividual strengths (red) across countries. Negative values indicate an advantage for women, whereas positive values indicate an advantage for men.

Two striking patterns emerged from our analysis. First, sex differences in mean scores were generally smaller—approximately half the size—and more variable than those in intraindividual strengths, which appear to be universal and consistent, particularly for literacy and numeracy (Figure 3). Second, sex differences in intraindividual strengths in literacy and numeracy were strongly and positively related at the country level.

Examining these patterns within countries and across age groups provides strong support for the previous analyses. Women universally demonstrated an advantage in literacy as an intraindividual strength, with coefficients ranging from −0.68 (95% CI = [−0.83, −0.54], *p* < .001) in Estonia for the 35–44 years age group to −0.20 (95% CI = [−0.34, −0.06], *p* = .006) in Croatia for the 55+ years age group. The only exception was once again the Slovak Republic, where a slight, non-significant advantage for men was observed among the youngest participants. However, non-significant differences favoring women were also found in a few countries (Korea, Poland, and Spain for the 16–24 years age group; in Croatia, Poland, and Singapore for the 25–34 years age group; in Poland and Spain for the 45–54 years age group; and in Poland and Portugal for those over 55 years).

Similarly, sex differences favoring men in numeracy as an intraindividual strength were also universal, meaning that across age groups, we did not observe a single country where women outperformed men in this strength. In New Zealand, among individuals aged 35–44 years, sex differences were particularly large, reaching 0.74 SDs (95% CI = [0.43, 1.04], *p* < .001). In contrast, the smallest sex differences in numeracy as an intraindividual strength (0.19, 95% CI = [0.02, 0.37], *p* = .03) were found among those aged 55+ years in Israel. However, these differences were not significant in the 24 years or younger age group in Poland, the Slovak Republic, Chile, Croatia, Ireland, Korea, Lithuania, Portugal, and Singapore; in the 25–34 years age group in Poland, the Slovak Republic, Chile, Croatia, Italy, Korea, and Singapore; in the 35–44 years age group in Croatia, Poland, Portugal, Singapore, Korea, and the Slovak Republic; in the 45–54 years age group in Poland, the Slovak Republic, Korea, and Singapore; and in the 55+ years age group in Croatia, Lithuania, Portugal, Poland, the Slovak Republic, and the Czech Republic.

In line with our hypotheses, sex differences in problem-solving as an intraindividual strength were generally not statistically significant across age groups and countries. However, there were exceptions, with either men or women having an advantage. The largest advantage for women was observed in Canada among individuals aged 24 years or younger (−0.34 SDs, 95% CI = [−0.60, −0.09], *p* = 0.008). Similar advantages were observed in Japan and Spain within the same age group, in Canada and New Zealand among individuals aged 35–44, and in Canada, Japan, the U.K., Switzerland, and Spain for the 45–54 and/or 55+ age groups (see the Supplementary Material for the full list of effect sizes).

Conversely, men performed better than women—slightly but significantly—in Chile (among those aged 24 years or younger), Lithuania, and Croatia (among those aged 35–44 years and 55+), with Chile showing the largest coefficient (0.24, 95% CI = [0.06, 0.42], *p* = 0.008).

**Figure 4:**
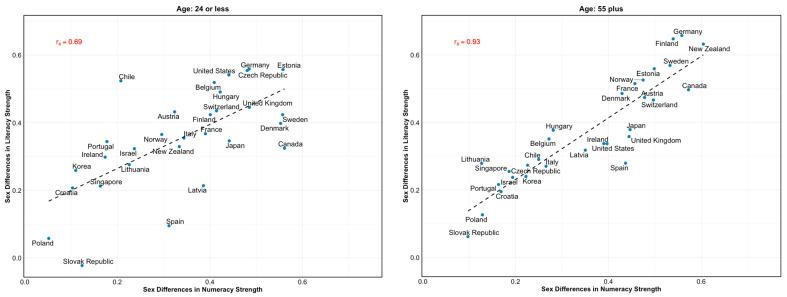
**Correlation Between sex differences in literacy and numeracy as intraindividual strengths by age group.** Spearman correlation (rs) between standardized sex differences in literacy (women > men) and numeracy (men > women) as intraindividual strengths. The lower (**left panel**) and higher (**right panel**) coefficients are presented. To aid interpretation, sex differences in literacy were multiplied by +1. See the Supplementary Material for a complete account of the age groups.

Notably, a strong correlation between sex differences in numeracy and literacy at the country level was also largely observed across age groups (Figure 4). Overall, a 1 SD increase in men’s advantage in numeracy was associated with a corresponding increase in women’s advantage in literacy—ranging from 0.91 SDs (95% CI = [0.75, 1.06], *p* < .001) in the 55+ years age group to 0.66 SDs (95% CI = [0.37, 0.95], *p* < .001) in the 24 years or younger age group.

## 4. Discussion

We used data from the second cycle of the PIAAC to examine sex differences in literacy, numeracy, and problem-solving as intraindividual strengths across 30 countries and five age groups. We found little to no evidence of an advantage for either men or women in the mean scores for literacy and problem-solving. When differences did emerge, they generally favored women and were small in magnitude. By contrast, a trivial but consistent advantage for men was observed in mean numeracy. Taken together, the results indicate substantial variation in sex differences by country and age group; however, when present, these differences were generally negligible.

In contrast, sex differences in intraindividual strengths were not only larger in magnitude than those in mean scores (approximately twice the size), but also more consistent; this was the case overall, across countries and age groups. In line with our first hypothesis, women outperformed men in literacy as an intraindividual strength, whereas men outperformed women in numeracy. These patterns were substantially universal, that is, found in each country and age group, with only a few exceptions. However, no clear advantage emerged for either sex in problem-solving as an intraindividual strength.

We found no support for our second hypothesis, as sex differences in intraindividual strengths did not appear to vary across age groups. This outcome suggests that these differences may remain consistent throughout the lifespan. One possible explanation is that, regardless of whether cognitive decline occurs, it may be evenly distributed across cognitive domains within individuals, thereby maintaining stable sex differences in intraindividual strengths. Another possibility is that the “age” grouping in PIAAC lacked sufficient high-end granularity. Specifically, the oldest category spans ages 55 to 65, which may still be too young to detect any statistically significant differential cognitive decline between men and women.

Notably, we found a strong and consistent positive association between sex differences in literacy and numeracy, both overall and across age groups. In other words, as the female advantage in literacy as an intraindividual strength increased at the country level, the male advantage in numeracy also increased, and by a similar magnitude. This surprising pattern aligns with research showing a close interrelation between mathematics and reading skills, suggesting that gains in one domain are often accompanied by gains in the other (Bergold et al., 2017). It is possible that efforts in many countries to boost girls’ performance in mathematics may have inadvertently strengthened their relative advantage in verbal domains instead (Balducci, 2023). In line with this prediction, Guiso et al. (2008) noted that the reduction in sex differences in mean mathematics scores observed in many countries was mostly due to an increase in mean reading scores for women.

Our findings largely support previous research, which reports small or nonexistent sex differences in reading and mathematics, but an advantage for females in verbal intraindividual strengths and an advantage for males in numeracy strengths (Balducci et al., 2024, 2025; Stoet & Geary, 2018). Our results add to those of previous studies, showing that these advantages are also found largely and universally throughout the lifespan.

According to expectancy-value theory, individuals with intraindividual strengths in numeracy should be more likely to enter STEM fields, whereas those with strengths in literacy should be more likely to pursue interpersonal occupations (e.g., Dekhtyar et al., 2018). Therefore, we propose that sex differences in intraindividual strengths contribute to the underrepresentation of women in STEM. This is not to suggest that intraindividual strengths are the sole explanation, as other factors, such as gender stereotypes (Moè et al., 2021), might also play a role. Nevertheless, intraindividual strengths are likely an important factor. Notably, the stability of these differences across age groups suggests that intraindividual strengths may have influenced career decisions not only among younger generations but also among later cohorts. Additionally, they could help explain career dropout, particularly among women who enter STEM fields despite having stronger verbal than numerical strengths—although this remains to be examined.

Based on this reasoning, intraindividual strengths pose a challenge to policies aimed at equalizing men’s and women’s participation in STEM, as career choices appear to be influenced by individual traits that, on average, might differ between the sexes. At the same time, the novelty of the concept and the fact that intraindividual strengths have not yet been incorporated into policies open new opportunities. For instance, focusing solely on raising the average numerical domain scores among girls and women might inadvertently steer them away from STEM fields, as this approach may further amplify their intraindividual strengths in verbal domains. A more effective strategy could involve identifying women with numerical strengths and actively encouraging them to pursue STEM careers. Similarly, supporting men with verbal strengths in exploring less traditionally sex-typed fields would also help promote equality of opportunity.

The findings of this study also have implications beyond the underrepresentation of women in STEM. In fact, another key inference is that, given the consistency and magnitude of sex differences in intraindividual strengths, future research on cognitive sex differences may benefit from shifting the focus away from mean scores—which typically show negligible differences—toward intraindividual patterns.

This study was descriptive in nature—we focused on outlining sex differences in intraindividual strengths without examining the variables that might be associated with their expression. Scholars have suggested that growing up with same-sex or opposite-sex siblings may have little impact, implying that these strengths may not be easily shaped by environmental factors (Fellman et al., 2021). However, caution is warranted, as the mechanisms underlying the development and expression of intraindividual strengths remain unclear at this stage. Future research should address this gap by exploring both societal and individual factors that may shape sex differences in intraindividual strength.

Given the descriptive nature of this study, we did not directly test expectancy-value theory, as it was beyond our research scope. However, previous research has substantially supported this theory and shown that individuals with numerical intraindividual strengths are more likely to pursue STEM fields, whereas those with verbal strengths are more likely to choose more interpersonal careers (Dekhtyar et al., 2018; Lubinski & Benbow, 2006; Stoet & Geary, 2018).

Future studies could build on current knowledge by directly examining the relationship between sex differences in intraindividual strengths and sex disparities in STEM participation. It would also be valuable to compare the predictive power of intraindividual strengths with other related measures, such as mean scores or ability tilt (i.e., one numerical vs. one verbal domain, such as numeracy minus literacy) (Wai et al., 2018), to determine which best explains the underrepresentation of women in STEM.

It is possible that sex differences in intraindividual strength were either underestimated or overestimated in this study. Tests such as the PIAAC are low-stakes assessments for participants, meaning that men and women might approach them differently, potentially exaggerating the observed sex differences. Conversely, the methodology used in the PIAAC may also conceal the true magnitude of these differences. For example, Wittmann (2005) noted that when sex differences are calculated using raw scores from the PISA—a test with a methodology similar to the PIAAC (see Section 2)—they appear nearly twice as large as those based on PVs. Future research should investigate whether the findings of this study can be replicated using other datasets.

Another limitation relates to the Literacy measurement used by PIAAC, which includes only reading proficiency and excludes writing skills. The ability to produce logical and relevant prose in response to a problem or issue (i.e., writing) can be regarded as a measure of the aptitude to formulate or express ideas. This specific skill might be distinct from the reading skills that are theorized to draw women away from STEM fields. In other words, the reading-only literacy measure used by PIAAC may not adequately capture the aspect of literacy that relates to decisions regarding STEM careers.

Lastly, previous research has shown that intraindividual academic strengths are more pronounced in more gender-equal countries, a phenomenon known as the gender equality paradox (Balducci et al., 2024; Stoet & Geary, 2018). Testing this paradox in our study setting posed several challenges. First, our small sample of countries, comprising mostly developed nations with relatively high gender equality (i.e., low variability), limited our ability to explore this phenomenon. Second, the impact of gender equality is likely nonlinear and may vary by age, suggesting that different indicators should have been used. However, the age groups we examined include individuals born up to nine years apart, making it difficult to pinpoint the exact period when gender equality should be measured. Although using age as a continuous variable would have been ideal, this information was missing for most individuals in our dataset. Despite these important caveats, Spearman correlations between sex differences in intraindividual strengths and the Global Gender Gap Index 2022 (the year when data collection for the second cycle of the PIAAC was largely conducted) support the gender equality paradox. Specifically, when considering the overall effect (i.e., not dividing the sample by age group), sex differences in both literacy (0.45, *p* = .01) and numeracy (0.47, *p* = .01) were wider in more gender-equal countries. However, future research should explore this paradox using a more robust approach.

In conclusion, sex differences in the mean scores for literacy, numeracy, and problem-solving appear to be trivial or nonexistent. However, when computed as intraindividual strengths, sex differences are consistent and much larger in magnitude. We recommend that researchers and policymakers consider this novel perspective, moving beyond mean scores to focus on intraindividual strengths and related patterns in the analysis of sex differences in cognitive domains, when addressing the underrepresentation of women in STEM.

## Data Availability

The dataset analyzed for this study is publicly available on the OECD’s official website: https://www.oecd.org/en/about/programmes/piaac.html (accessed on 4 April 2025).

## Supplementary Materials

The following supporting information can be downloaded at https://www.mdpi.com/article/10.3390/jintelligence14010012/s1. Table S1: distribution of respondents by country and age groups; Table S2: standardized sex differences (men–women) in literacy as mean scores by country and age groups; Table S3: standardized sex differences (men–women) in numeracy as mean scores by country and age groups; Table S4: standardized sex differences (men–women) in problem-solving as mean scores by country and age groups; Table S5: standardized sex differences (men–women) in literacy as intraindividual strengths by country and age groups; Table S6: standardized sex differences (men–women) in numeracy as intraindividual strengths by country and age groups; Table S7: standardized sex differences (men–women) in problem-solving as intraindividual strengths by country and age groups; Figure S1: correlation between sex differences in literacy and numeracy as intraindividual strengths.
