# Supplementary material for: Beyond Mean Scores: Sex Differences in Literacy, Numeracy, and Problem-Solving as Intraindividual Strengths Across Age Groups
Source: J Intell. 2026 Jan 6;14(1):12. doi: 10.3390/jintelligence14010012 (PMC12843321; doi:10.3390/jintelligence14010012)
Supplement: Supplementary file 1 [file jintelligence-14-00012-s001.zip › jintelligence-4056176-supplementary.pdf]

Supplementary material

Table S1. Distribution of respondents by country and age groups.

Serial	Country	Age categories					Total
		24 or less	25-34	35-44	45-54	55 plus	
1	Austria	722 (3.11%)	850 (2.91%)	898 (2.71%)	902 (2.70%)	1,193 (3.14%)	4,565 (2.91%)
2	Belgium	678 (2.92%)	679 (2.33%)	768 (2.32%)	818 (2.45%)	966 (2.54%)	3,909 (2.49%)
3	Canada	1,381 (5.95%)	1,940 (6.65%)	2,487 (7.52%)	2,469 (7.40%)	3,420 (8.99%)	11,697 (7.46%)
4	Chile	772 (3.32%)	1,145 (3.92%)	975 (2.95%)	848 (2.54%)	986 (2.59%)	4,726 (3.01%)
5	Croatia	687 (2.96%)	800 (2.74%)	806 (2.44%)	900 (2.70%)	1,123 (2.95%)	4,316 (2.75%)
6	Czech Republic	778 (3.35%)	910 (3.12%)	1,056 (3.19%)	1,167 (3.50%)	1,146 (3.01%)	5,057 (3.22%)
7	Denmark	634 (2.73%)	980 (3.36%)	1,094 (3.31%)	1,139 (3.41%)	1,220 (3.21%)	5,067 (3.23%)
8	Estonia	883 (3.80%)	1,183 (4.05%)	1,491 (4.51%)	1,500 (4.50%)	1,608 (4.23%)	6,665 (4.25%)
9	Finland	494 (2.13%)	745 (2.55%)	866 (2.62%)	815 (2.44%)	1,141 (3.00%)	4,061 (2.59%)
10	France	1,064 (4.58%)	1,015 (3.48%)	1,339 (4.05%)	1,456 (4.36%)	1,558 (4.10%)	6,432 (4.10%)
11	Germany	746 (3.21%)	963 (3.30%)	983 (2.97%)	915 (2.74%)	1,186 (3.12%)	4,793 (3.05%)
12	Hungary	691 (2.98%)	813 (2.79%)	946 (2.86%)	1,123 (3.37%)	991 (2.61%)	4,564 (2.91%)
13	Ireland	410 (1.77%)	618 (2.12%)	1,038 (3.14%)	897 (2.69%)	889 (2.34%)	3,852 (2.46%)
14	Israel	1,500 (6.46%)	1,445 (4.95%)	1,180 (3.57%)	1,047 (3.14%)	920 (2.42%)	6,092 (3.88%)
15	Italy	1,324 (5.70%)	979 (3.35%)	753 (2.28%)	911 (2.73%)	880 (2.31%)	4,847 (3.09%)

16	Japan	674 (2.90%)	821 (2.81%)	1,097 (3.32%)	1,305 (3.91%)	1,268 (3.33%)	5,165 (3.29%)
17	Korea	607 (2.61%)	1,098 (3.76%)	1,382 (4.18%)	1,399 (4.19%)	1,712 (4.50%)	6,198 (3.95%)
18	Latvia	553 (2.38%)	904 (3.10%)	1,362 (4.12%)	1,515 (4.54%)	2,229 (5.86%)	6,563 (4.18%)
19	Lithuania	684 (2.95%)	1,176 (4.03%)	1,143 (3.45%)	1,282 (3.84%)	1,901 (5.00%)	6,186 (3.94%)
20	New Zealand	1,260 (5.43%)	1,016 (3.48%)	1,056 (3.19%)	986 (2.96%)	1,041 (2.74%)	5,359 (3.42%)
21	Norway	610 (2.63%)	731 (2.50%)	769 (2.32%)	765 (2.29%)	857 (2.25%)	3,732 (2.38%)
22	Poland	778 (3.35%)	982 (3.36%)	1,138 (3.44%)	1,010 (3.03%)	1,106 (2.91%)	5,014 (3.20%)
23	Portugal	489 (2.11%)	594 (2.03%)	701 (2.12%)	735 (2.20%)	641 (1.69%)	3,160 (2.01%)
24	Singapore	755 (3.25%)	1,110 (3.80%)	1,063 (3.21%)	976 (2.93%)	1,107 (2.91%)	5,011 (3.19%)
25	Slovak Republic	748 (3.22%)	992 (3.40%)	1,238 (3.74%)	1,170 (3.51%)	1,090 (2.87%)	5,238 (3.34%)
26	Spain	648 (2.79%)	1,009 (3.46%)	1,321 (3.99%)	1,506 (4.51%)	1,387 (3.65%)	5,871 (3.74%)
27	Sweden	606 (2.61%)	641 (2.20%)	718 (2.17%)	685 (2.05%)	752 (1.98%)	3,402 (2.17%)
28	Switzerland	967 (4.16%)	1,269 (4.35%)	1,424 (4.30%)	1,448 (4.34%)	1,540 (4.05%)	6,648 (4.24%)
29	United Kingdom	491 (2.11%)	1,017 (3.48%)	1,198 (3.62%)	1,007 (3.02%)	1,228 (3.23%)	4,941 (3.15%)
30	United States	591 (2.54%)	766 (2.62%)	802 (2.42%)	665 (1.99%)	941 (2.47%)	3,765 (2.40%)
	Total	23,225 (14.80%)	29,191 (18.61%)	33,092 (21.09%)	33,361 (21.26%)	38,027 (24.24%)	156,896 (100%)

Table S2. Standardized sex differences (men–women) in literacy as mean scores by country and age group.

		Sex differences in literacy as mean scores					
			Age categories				
		Overall	24 or less	25-34	35-44	45-54	55 plus
Serial	Country	Coefficient (female)	Coefficient (female)	Coefficient (female)	Coefficient (female)	Coefficient (female)	Coefficient (female)
1	Austria	-0.00	0.04	–0.14	0.01	–0.00	0.04
		(0.03)	(0.06)	(0.08)	(0.08)	(0.07)	(0.06)
2	Belgium	-0.01	–0.15*	0.00	–0.11	0.08	0.06
		(0.03)	(0.07)	(0.07)	(0.07)	(0.07)	(0.07)
3	Canada	0.01	0.04	–0.03	0.15*	0.05	–0.17*
		(0.04)	(0.08)	(0.08)	(0.07)	(0.10)	(0.07)
4	Chile	-0.01	0.02	–0.04	0.13	–0.19	–0.08
		(0.05)	(0.08)	(0.08)	(0.09)	(0.12)	(0.07)
5	Croatia	-0.13***	–0.13	–0.18*	–0.15*	–0.12	–0.12
		(0.03)	(0.09)	(0.08)	(0.07)	(0.08)	(0.06)
6	Czech Republic	-0.03	–0.07	–0.15	0.03	0.01	–0.03
		(0.04)	(0.11)	(0.12)	(0.09)	(0.08)	(0.09)
7	Denmark	-0.05	–0.07	0.07	–0.10	–0.11*	–0.05
		(0.03)	(0.07)	(0.08)	(0.06)	(0.05)	(0.06)
8	Estonia	-0.19***	–0.27***	–0.13*	–0.24***	–0.27***	–0.20***
		(0.02)	(0.05)	(0.06)	(0.05)	(0.05)	(0.05)
9	Finland	-0.12**	–0.11	–0.07	–0.21*	–0.03	–0.20**
		(0.03)	(0.09)	(0.08)	(0.09)	(0.07)	(0.06)
10	France	-0.06*	–0.09	–0.03	–0.10	0.06	–0.07
		(0.03)	(0.05)	(0.07)	(0.06)	(0.06)	(0.06)
11	Germany	-0.07*	–0.02	–0.08	–0.11	–0.12	–0.05
		(0.03)	(0.07)	(0.08)	(0.07)	(0.07)	(0.06)
12	Hungary	-0.12**	–0.11	–0.08	–0.11	–0.15*	–0.19***
		(0.03)	(0.07)	(0.08)	(0.06)	(0.06)	(0.06)
13	Ireland	-0.07	–0.13	–0.01	0.00	–0.11	–0.15
		(0.04)	(0.10)	(0.11)	(0.09)	(0.10)	(0.09)
14	Israel	-0.10**	–0.13*	–0.13*	–0.20**	–0.07	0.05
		(0.03)	(0.05)	(0.06)	(0.07)	(0.08)	(0.09)
15	Italy	-0.06	–0.06	–0.06	–0.05	–0.15*	0.00
		(0.03)	(0.07)	(0.10)	(0.08)	(0.07)	(0.07)

16	Japan	-0.00<br>(0.03)	0.03<br>(0.09)	–0.03<br>(0.07)	–0.09<br>(0.07)	0.01<br>(0.07)	0.04<br>(0.07)
17	Korea	0.03<br>(0.03)	–0.08<br>(0.09)	0.02<br>(0.07)	–0.01<br>(0.06)	–0.04<br>(0.06)	0.10<br>(0.05)
18	Latvia	-0.07*<br>(0.04)	–0.17<br>(0.11)	–0.06<br>(0.09)	–0.12<br>(0.07)	–0.08<br>(0.08)	–0.12<br>(0.06)
19	Lithuania	-0.09*<br>(0.04)	0.07<br>(0.11)	–0.05<br>(0.08)	–0.13<br>(0.08)	–0.12<br>(0.08)	–0.23***<br>(0.06)
20	New Zealand	-0.20**<br>(0.06)	–0.23*<br>(0.12)	–0.30<br>(0.16)	–0.21<br>(0.14)	–0.02<br>(0.14)	–0.22<br>(0.11)
21	Norway	-0.08*<br>(0.04)	–0.14<br>(0.08)	–0.01<br>(0.07)	–0.14<br>(0.09)	0.01<br>(0.10)	–0.12<br>(0.08)
22	Poland	-0.06*<br>(0.03)	0.04<br>(0.08)	–0.15*<br>(0.07)	–0.08<br>(0.06)	–0.05<br>(0.07)	–0.07 (<br>0.07)
23	Portugal	0.04<br>(0.04)	0.02<br>(0.10)	–0.07<br>(0.12)	–0.05<br>(0.11)	0.10<br>(0.11)	0.07<br>(0.09)
24	Singapore	0.07*<br>(0.03)	–0.11*<br>(0.06)	–0.06<br>(0.06)	0.16*<br>(0.06)	0.13<br>(0.07)	0.12<br>(0.07)
25	Slovak Republic	-0.01<br>(0.03)	0.07<br>(0.10)	–0.04<br>(0.09)	0.03<br>(0.07)	–0.03<br>(0.08)	–0.08<br>(0.08)
26	Spain	0.04<br>(0.03)	0.12<br>(0.09)	0.02<br>(0.09)	0.01<br>(0.07)	–0.04<br>(0.06)	0.10<br>(0.07)
27	Sweden	-0.05<br>(0.04)	–0.20*<br>(0.10)	–0.02<br>(0.12)	–0.06<br>(0.11)	0.08<br>(0.07)	0.10<br>(0.08)
28	Switzerland	0.05<br>(0.04)	–0.10<br>(0.07)	0.06<br>(0.08)	–0.01<br>(0.08)	0.11<br>(0.07)	0.10<br>(0.06)
29	United Kingdom	0.04<br>(0.04)	–0.06<br>(0.08)	0.03<br>(0.08)	0.10<br>(0.09)	0.13<br>(0.11)	-0.01<br>(0.08)
30	United States	-0.05<br>(0.03)	–0.15<br>(0.09)	0.13<br>(0.10)	–0.05<br>(0.10)	–0.09<br>(0.10)	–0.10<br>(0.09)

Standard errors in parentheses.
* $p < 0.05$, ** $p < 0.01$, *** $p < 0.001$.

Table S3. Standardized sex differences (men–women) in numeracy as mean scores by country and age groups.

		Sex differences in numeracy as mean scores					
			Age categories				
		Overall	24 or less	25-34	35-44	45-54	55 plus
Serial	Country	Coefficient (female)	Coefficient (female)	Coefficient (female)	Coefficient (female)	Coefficient (female)	Coefficient (female)
1	Austria	0.25***<br>(0.03)	0.24**<br>(0.07)	0.14*<br>(0.07)	0.27***<br>(0.08)	0.28***<br>(0.08)	0.29***<br>(0.06)
2	Belgium	0.18***<br>(0.03)	0.10<br>(0.07)	0.22**<br>(0.08)	0.09<br>(0.07)	0.26***<br>(0.07)	0.22**<br>(0.07)
3	Canada	0.29***<br>(0.03)	0.29***<br>(0.08)	0.28***<br>(0.08)	0.42***<br>(0.07)	0.33***<br>(0.09)	0.13*<br>(0.07)
4	Chile	0.15**<br>(0.05)	0.21**<br>(0.08)	0.11<br>(0.07)	0.27**<br>(0.09)	-0.04<br>(0.11)	0.05<br>(0.08)
5	Croatia	–0.02<br>(0.03)	-0.04<br>(0.08)	-0.09<br>(0.07)	-0.01<br>(0.06)	-0.01<br>(0.08)	-0.01<br>(0.07)
6	Czech Republic	0.21***<br>(0.04)	0.24*<br>(0.12)	0.13<br>(0.12)	0.27**<br>(0.09)	0.26***<br>(0.08)	0.12<br>(0.10)
7	Denmark	0.15***<br>(0.03)	0.13*<br>(0.07)	0.25***<br>(0.07)	0.09<br>(0.06)	0.12*<br>(0.05)	0.14*<br>(0.06)
8	Estonia	0.13***<br>(0.03)	0.03<br>(0.06)	0.20***<br>(0.06)	0.14**<br>(0.05)	0.05<br>(0.05)	0.09<br>(0.06)
9	Finland	0.18***<br>(0.03)	0.11<br>(0.03)	0.22**<br>(0.08)	0.13<br>(0.09)	0.26**<br>(0.08)	0.12<br>(0.07)
10	France	0.18***<br>(0.03)	0.11*<br>(0.05)	0.20**<br>(0.07)	0.16**<br>(0.06)	0.18**<br>(0.07)	0.19**<br>(0.07)
11	Germany	0.21***<br>(0.03)	0.24***<br>(0.07)	0.21*<br>(0.08)	0.17*<br>(0.08)	0.16*<br>(0.07)	0.25***<br>(0.07)
12	Hungary	0.08*<br>(0.03)	0.13<br>(0.03)	0.17*<br>(0.07)	0.09<br>(0.07)	0.01<br>(0.07)	-0.01<br>(0.06)
13	Ireland	0.19***<br>(0.05)	0.02<br>(0.11)	0.24*<br>(0.10)	0.32**<br>(0.10)	0.23*<br>(0.10)	0.09<br>(0.09)
14	Israel	0.10**<br>(0.03)	0.05<br>(0.06)	0.08<br>(0.07)	0.04<br>(0.08)	0.13<br>(0.08)	0.19*<br>(0.08)
15	Italy	0.12***<br>(0.03)	0.14<br>(0.08)	0.10<br>(0.09)	0.17*<br>(0.09)	0.03<br>(0.08)	0.15<br>(0.08)

16	Japan	0.24***<br>(0.04)	0.25**<br>(0.09)	0.23**<br>(0.08)	0.16*<br>(0.07)	0.25**<br>(0.07)	0.27***<br>(0.08)
17	Korea	0.15***<br>(0.03)	0.03<br>(0.10)	0.16*<br>(0.07)	0.09<br>(0.06)	0.08<br>(0.06)	0.24***<br>(0.06)
18	Latvia	0.14***<br>(0.04)	0.01<br>(0.12)	0.16<br>(0.10)	0.11<br>(0.07)	0.18*<br>(0.08)	0.08<br>(0.07)
19	Lithuania	0.09*<br>(0.04)	0.23*<br>(0.10)	0.16*<br>(0.08)	0.09<br>(0.07)	0.08<br>(0.08)	-0.10<br>(0.07)
20	New Zealand	0.08<br>(0.06)	-0.06<br>(0.14)	-0.02<br>(0.14)	0.13<br>(0.11)	0.27*<br>(0.13)	0.11<br>(0.11)
21	Norway	0.19***<br>(0.04)	0.05<br>(0.08)	0.22**<br>(0.07)	0.18*<br>(0.09)	0.30**<br>(0.10)	0.17*<br>(0.08)
22	Poland	0.002<br>(0.03)	0.08<br>(0.08)	-0.12<br>(0.07)	0.01<br>(0.07)	0.00<br>(0.07)	0.02<br>(0.06)
23	Portugal	0.17***<br>(0.04)	0.16<br>(0.09)	0.10<br>(0.13)	0.07<br>(0.11)	0.24*<br>(0.11)	0.18<br>(0.10)
24	Singapore	0.16***<br>(0.03)	-0.01<br>(0.07)	0.03<br>(0.06)	0.25***<br>(0.06)	0.22**<br>(0.07)	0.24***<br>(0.07)
25	Slovak Republic	0.03<br>(0.03)	0.10<br>(0.09)	0.00<br>(0.09)	0.06<br>(0.07)	0.04<br>(0.08)	-0.03<br>(0.07)
26	Spain	0.19***<br>(0.03)	0.24**<br>(0.09)	0.16<br>(0.09)	0.17*<br>(0.07)	0.08<br>(0.06)	0.31***<br>(0.07)
27	Sweden	0.22***<br>(0.04)	0.06<br>(0.09)	0.27*<br>(0.12)	0.20<br>(0.10)	0.36***<br>(0.06)	0.21**<br>(0.08)
28	Switzerland	0.31***<br>(0.04)	0.13*<br>(0.06)	0.35***<br>(0.09)	0.25**<br>(0.09)	0.34***<br>(0.07)	0.37***<br>(0.0.6)
29	United Kingdom	0.28***<br>(0.04)	0.19<br>(0.11)	0.29**<br>(0.08)	0.33***<br>(0.08)	0.38**<br>(0.11)	0.21*<br>(0.08)
30	United States	0.12**<br>(0.03)	0.07<br>(0.08)	0.28**<br>(0.08)	0.13<br>(0.11)	0.03<br>(0.11)	0.06<br>(0.10)

Standard errors in parentheses.
* $p < 0.05$, ** $p < 0.01$, *** $p < 0.001$.

Table S4. Standardized sex differences (men–women) in problem-solving as mean scores by country and age groups.

		Sex differences in problem-solving as mean scores					
			Age categories				
		Overall	24 or less	25-34	35-44	45-54	55 plus
Serial	Country	Coefficient (female)	Coefficient (female)	Coefficient (female)	Coefficient (female)	Coefficient (female)	Coefficient (female)
1	Austria	0.13*** (0.04)	0.16* (0.07)	0.04 (0.08)	0.14 (0.08)	0.14 (0.07)	0.13* (0.06)
2	Belgium	0.07* (0.03)	0.01 (0.08)	0.09 (0.07)	-0.05 (0.08)	0.15* (0.07)	0.15* (0.07)
3	Canada	0.06 (0.04)	0.02 (0.08)	0.04 (0.08)	0.18* (0.08)	0.11 (0.09)	-0.07 (0.07)
4	Chile	0.12* (0.05)	0.24** (0.09)	0.12 (0.08)	0.19* (0.09)	-0.10 (0.13)	0.00 (0.07)
5	Croatia	-0.03 (0.03)	-0.03 (0.08)	-0.10 (0.08)	0.01 (0.07)	-0.05 (0.07)	-0.05 (0.06)
6	Czech Republic	0.09 (0.05)	0.07 (0.11)	-0.03 (0.11)	0.17 (0.09)	0.13 (0.08)	0.05 (0.10)
7	Denmark	0.05 (0.03)	-0.03 (0.07)	0.15 (0.08)	0.01 (0.06)	0.01 (0.05)	0.05 (0.06)
8	Estonia	-0.02 (0.02)	-0.12* (0.06)	0.07 (0.06)	-0.07 (0.05)	-0.12* (0.05)	-0.03 (0.05)
9	Finland	0.06 (0.04)	0.01 (0.10)	0.09 (0.08)	-0.01 (0.09)	0.13 (0.08)	0.02 (0.07)
10	France	0.05 (0.03)	-0.01 (0.06)	0.05 (0.07)	0.02 (0.06)	0.06 (0.07)	0.06 (0.06)
11	Germany	0.06 (0.03)	0.10 (0.07)	0.05 (0.09)	0.01 (0.07)	-0.00 (0.07)	0.10 (0.06)
12	Hungary	0.00 (0.03)	0.01 (0.08)	0.05 (0.08)	0.03 (0.07)	-0.07 (0.07)	-0.06 (0.06)
13	Ireland	0.04 (0.06)	0.02 (0.12)	0.09 (0.11)	0.16 (0.10)	-0.04 (0.09)	-0.05 (0.09)
14	Israel	0.02 (0.03)	-0.00 (0.06)	-0.01 (0.07)	-0.07 (0.07)	0.05 (0.08)	0.11 (0.09)
15	Italy	0.06 (0.04)	0.04 (0.08)	0.05 (0.11)	0.14 (0.10)	-0.02 (0.09)	0.06 (0.07)

16	Japan	0.04<br>(0.03)	0.05<br>(0.10)	0.03<br>(0.08)	-0.04<br>(0.07)	0.03<br>(0.07)	0.07<br>(0.08)
17	Korea	0.12***<br>(0.03)	0.05<br>(0.09)	0.16*<br>(0.07)	0.07<br>(0.06)	0.07<br>(0.06)	0.15**<br>(0.06)
18	Latvia	0.03<br>(0.04)	-0.16<br>(0.13)	0.01<br>(0.09)	0.01<br>(0.07)	0.08<br>(0.08)	-0.04<br>(0.06)
19	Lithuania	0.09*<br>(0.04)	0.14<br>(0.11)	0.12<br>(0.09)	0.12<br>(0.08)	0.05<br>(0.08)	-0.06<br>(0.07)
20	New Zealand	-0.08<br>(0.06)	-0.14<br>(0.14)	-0.10<br>(0.14)	-0.14<br>(0.12)	0.03<br>(0.15)	-0.05<br>(0.11)
21	Norway	0.04<br>(0.04)	-0.02<br>(0.08)	0.09<br>(0.08)	-0.01<br>(0.09)	0.10<br>(0.09)	0.02<br>(0.08)
22	Poland	-0.02<br>(0.04)	0.05<br>(0.09)	-0.08<br>(0.07)	-0.02<br>(0.07)	-0.04<br>(0.08)	-0.03<br>(0.07)
23	Portugal	0.15***<br>(0.04)	0.15<br>(0.09)	0.09<br>(0.13)	0.12<br>(0.10)	0.19<br>(0.10)	0.13<br>(0.09)
24	Singapore	0.12***<br>(0.03)	-0.04<br>(0.06)	-0.01<br>(0.06)	0.22**<br>(0.07)	0.18**<br>(0.06)	0.17**<br>(0.06)
25	Slovak Republic	-0.01<br>(0.04)	-0.01<br>(0.11)	-0.04<br>(0.08)	0.02<br>(0.06)	0.02<br>(0.08)	-0.06<br>(0.08)
26	Spain	0.04<br>(0.03)	0.05<br>(0.10)	0.08<br>(0.09)	0.01<br>(0.07)	-0.06<br>(0.06)	0.10<br>(0.07)
27	Sweden	0.03<br>(0.04)	-0.15<br>(0.10)	0.07<br>(0.12)	-0.01<br>(0.12)	0.19**<br>(0.07)	0.03<br>(0.08)
28	Switzerland	0.12**<br>(0.04)	0.00<br>(0.07)	0.14<br>(0.08)	0.06<br>(0.09)	0.18*<br>(0.08)	0.17**<br>(0.06)
29	United Kingdom	0.10*<br>(0.04)	0.02<br>(0.10)	0.10<br>(0.08)	0.18*<br>(0.09)	0.17<br>(0.11)	0.04<br>(0.08)
30	United States	0.04<br>(0.04)	-0.01<br>(0.09)	0.23**<br>(0.09)	0.03<br>(0.10)	-0.03<br>(0.10)	-0.05<br>(0.08)

Standard errors in parentheses.
* $p < 0.05$, ** $p < 0.01$, *** $p < 0.001$.

Table S5. Standardized sex differences (men–women) in literacy as intraindividual strengths by country and age groups.

		Sex differences in literacy as IIS					
			Age categories				
		Overall	24 or less	25-34	35-44	45-54	55 plus
Serial	Country	Coefficient (female)	Coefficient (female)	Coefficient (female)	Coefficient (female)	Coefficient (female)	Coefficient (female)
1	Austria	-0.52*** (0.03)	-0.43*** (0.07)	-0.61*** (0.07)	-0.52*** (0.06)	-0.57*** (0.06)	-0.47*** (0.05)
2	Belgium	-0.39*** (0.03)	-0.52*** (0.06)	-0.44*** (0.06)	-0.33** (0.06)	-0.35*** (0.05)	-0.35*** (0.05)
3	Canada	-0.46*** (0.03)	-0.32** (0.09)	-0.51*** (0.09)	-0.44*** (0.07)	-0.47*** (0.07)	-0.50*** (0.06)
4	Chile	-0.36*** (0.04)	-0.52*** (0.09)	-0.41*** (0.07)	-0.31* (0.08)	-0.28* (0.08)	-0.29* (0.07)
5	Croatia	-0.22*** (0.03)	-0.21* (0.07)	-0.16 (0.06)	-0.34*** (0.06)	-0.20* (0.06)	-0.20** (0.06)
6	Czech Republic	-0.44*** (0.03)	-0.55*** (0.08)	-0.47*** (0.07)	-0.49*** (0.07)	-0.46*** (0.06)	-0.27* (0.06)
7	Denmark	-0.49*** (0.03)	-0.40*** (0.08)	-0.45*** (0.07)	-0.51*** (0.06)	-0.59*** (0.05)	-0.49*** (0.05)
8	Estonia	-0.61*** (0.02)	-0.56*** (0.05)	-0.67*** (0.04)	-0.68*** (0.04)	-0.58*** (0.04)	-0.56*** (0.04)
9	Finland	-0.60*** (0.03)	-0.42*** (0.07)	-0.59*** (0.06)	-0.68*** (0.05)	-0.58*** (0.06)	-0.65*** (0.05)
10	France	-0.46*** (0.02)	-0.37*** (0.04)	-0.41*** (0.05)	-0.51*** (0.04)	-0.48*** (0.04)	-0.52*** (0.04)
11	Germany	-0.59*** (0.02)	-0.56*** (0.05)	-0.59*** (0.05)	-0.56*** (0.05)	-0.56*** (0.05)	-0.66*** (0.04)
12	Hungary	-0.41*** (0.02)	-0.49*** (0.06)	-0.50*** (0.05)	-0.45*** (0.05)	-0.30** (0.04)	-0.38*** (0.05)
13	Ireland	-0.40*** (0.03)	-0.30* (0.09)	-0.39** (0.08)	-0.52*** (0.07)	-0.44*** (0.07)	-0.44** (0.07)
14	Israel	-0.33*** (0.03)	-0.32*** (0.06)	-0.34*** (0.06)	-0.38*** (0.06)	-0.36*** (0.06)	-0.24** (0.06)
15	Italy	-0.35*** (0.03)	-0.35** (0.07)	-0.31** (0.08)	-0.48*** (0.08)	-0.35** (0.07)	-0.27** (0.07)

16	Japan	-0.39*** (0.02)	-0.35** (0.06)	-0.45*** (0.06)	-0.41*** (0.05)	-0.38*** (0.04)	-0.38*** (0.04)
17	Korea	-0.25*** (0.02)	-0.26 (0.07)	-0.33** (0.05)	-0.21* (0.04)	-0.24** (0.05)	-0.24** (0.04)
18	Latvia	-0.36*** (0.03)	-0.21* (0.10)	-0.36*** (0.08)	-0.39*** (0.07)	-0.49*** (0.06)	-0.32*** (0.06)
19	Lithuania	-0.36*** (0.03)	-0.28* (0.08)	-0.40*** (0.06)	-0.47*** (0.07)	-0.38*** (0.06)	-0.28*** (0.05)
20	New Zealand	-0.52* (0.06)	-0.33** (0.13)	-0.59*** (0.14)	-0.52*** (0.16)	-0.49** (0.11)	-0.63*** (0.10)
21	Norway	-0.48*** (0.03)	-0.37*** (0.06)	-0.41*** (0.06)	-0.57*** (0.06)	-0.50*** (0.06)	-0.53*** (0.05)
22	Poland	-0.10* (0.02)	-0.06 (0.06)	-0.08 (0.05)	-0.13 (0.05)	-0.06 (0.05)	-0.13 (0.05)
23	Portugal	-0.32*** (0.04)	-0.34* (0.08)	-0.40** (0.08)	-0.35** (0.08)	-0.32** (0.09)	-0.22 (0.08)
24	Singapore	-0.21*** (0.02)	-0.21** (0.05)	-0.17 (0.05)	-0.23* (0.05)	-0.20* (0.05)	-0.26** (0.05)
25	Slovak Republic	-0.05 (0.03)	0.02 (0.08)	-0.04 (0.07)	-0.02 (0.05)	-0.13 (0.06)	-0.06 (0.07)
26	Spain	-0.19*** (0.03)	-0.10 (0.07)	-0.23* (0.06)	-0.21* (0.05)	-0.12 (0.05)	-0.28** (0.06)
27	Sweden	-0.50*** (0.03)	-0.42*** (0.08)	-0.53*** (0.08)	-0.43*** (0.09)	-0.56*** (0.07)	-0.57*** (0.06)
28	Switzerland	-0.46*** (0.02)	-0.44*** (0.06)	-0.51*** (0.05)	-0.44*** (0.06)	-0.42*** (0.04)	-0.47*** (0.05)
29	United Kingdom	-0.42*** (0.03)	-0.45*** (0.09)	-0.45*** (0.07)	-0.43*** (0.06)	-0.43*** (0.06)	-0.36*** (0.06)
30	United States	-0.39*** (0.03)	-0.54*** (0.08)	-0.40*** (0.08)	-0.39*** (0.07)	-0.27* (0.08)	-0.34*** (0.07)

Standard errors in parentheses.
* $p < 0.05$, ** $p < 0.01$, *** $p < 0.001$.

Table S6. Standardized sex differences (men–women) in numeracy as intraindividual strengths by country and age groups.

		Sex differences in numeracy as IIS					
			Age categories				
		Overall	24 or less	25-34	35-44	45-54	55 plus
Serial	Country	Coefficient (female)	Coefficient (female)	Coefficient (female)	Coefficient (female)	Coefficient (female)	Coefficient (female)
1	Austria	0.44*** (0.03)	0.32*** (0.07)	0.46*** (0.07)	0.45*** (0.06)	0.50*** (0.06)	0.48*** (0.06)
2	Belgium	0.37*** (0.03)	0.41*** (0.06)	0.42*** (0.06)	0.42*** (0.06)	0.33*** (0.06)	0.27*** (0.05)
3	Canada	0.56*** (0.04)	0.56*** (0.10)	0.60*** (0.08)	0.54*** (0.08)	0.54*** (0.07)	0.57*** (0.06)
4	Chile	0.23*** (0.04)	0.21 (0.08)	0.19 (0.07)	0.26* (0.06)	0.29* (0.09)	0.25* (0.07)
5	Croatia	0.14** (0.03)	-0.10 (0.08)	-0.13 (0.07)	-0.15 (0.06)	-0.18* (0.06)	-0.17 (0.05)
6	Czech Republic	0.35*** (0.03)	0.48*** (0.07)	0.45*** (0.07)	0.33*** (0.06)	0.37*** (0.06)	0.23 (0.06)
7	Denmark	0.46*** (0.03)	0.55*** (0.08)	0.40*** (0.07)	0.42*** (0.05)	0.53*** (0.05)	0.43*** (0.05)
8	Estonia	0.56*** (0.02)	0.56*** (0.04)	0.54*** (0.04)	0.69*** (0.04)	0.59*** (0.04)	0.50*** (0.04)
9	Finland	0.52*** (0.03)	0.40*** (0.07)	0.53*** (0.06)	0.61*** (0.05)	0.51*** (0.06)	0.54*** (0.05)
10	France	0.43*** (0.02)	0.39*** (0.04)	0.43*** (0.05)	0.48*** (0.04)	0.44*** (0.04)	0.46*** (0.04)
11	Germany	0.52*** (0.02)	0.48*** (0.06)	0.52*** (0.06)	0.52*** (0.05)	0.54*** (0.05)	0.56*** (0.04)
12	Hungary	0.33*** (0.02)	0.42*** (0.05)	0.41*** (0.06)	0.32*** (0.06)	0.30*** (0.04)	0.28*** (0.05)
13	Ireland	0.41*** (0.03)	0.17 (0.09)	0.39*** (0.08)	0.45*** (0.06)	0.61*** (0.06)	0.39*** (0.06)
14	Israel	0.26*** (0.03)	0.24** (0.05)	0.29*** (0.06)	0.35*** (0.06)	0.27*** (0.06)	0.19* (0.06)
15	Italy	0.28*** (0.03)	0.34** (0.07)	0.25 (0.07)	0.29* (0.08)	0.28** (0.07)	0.27* (0.07)

16	Japan	0.47***<br>(0.02)	0.44***<br>(0.06)	0.51***<br>(0.06)	0.49***<br>(0.05)	0.47***<br>(0.04)	0.45***<br>(0.04)
17	Korea	0.15**<br>(0.02)	0.11<br>(0.07)	0.15<br>(0.05)	0.14<br>(0.04)	0.14<br>(0.04)	0.22**<br>(0.04)
18	Latvia	0.34***<br>(0.03)	0.39**<br>(0.10)	0.40**<br>(0.08)	0.35***<br>(0.07)	0.38***<br>(0.07)	0.35***<br>(0.05)
19	Lithuania	0.18***<br>(0.03)	0.23<br>(0.09)	0.24*<br>(0.06)	0.19*<br>(0.06)	0.24**<br>(0.06)	0.13<br>(0.05)
20	New Zealand	0.55***<br>(0.06)	0.33**<br>(0.08)	0.45**<br>(0.13)	0.74***<br>(0.13)	0.64***<br>(0.16)	0.61***<br>(0.09)
21	Norway	0.45***<br>(0.03)	0.30**<br>(0.06)	0.37***<br>(0.06)	0.56***<br>(0.06)	0.50***<br>(0.05)	0.48***<br>(0.05)
22	Poland	0.08<br>(0.03)	0.05<br>(0.07)	0.03<br>(0.06)	0.11<br>(0.05)	0.09<br>(0.05)	0.13<br>(0.05)
23	Portugal	0.16**<br>(0.04)	0.18<br>(0.08)	0.21*<br>(0.08)	0.10<br>(0.07)	0.21*<br>(0.07)	0.16<br>(0.08)
24	Singapore	0.14*<br>(0.02)	0.16<br>(0.05)	0.15<br>(0.05)	0.10<br>(0.05)	0.11<br>(0.05)	0.19*<br>(0.05)
25	Slovak Republic	0.08<br>(0.03)	0.12<br>(0.07)	0.09<br>(0.07)	0.05<br>(0.06)	0.09<br>(0.06)	0.10<br>(0.08)
26	Spain	0.32***<br>(0.03)	0.31*<br>(0.07)	0.22*<br>(0.06)	0.35***<br>(0.05)	0.28***<br>(0.05)	0.44***<br>(0.05)
27	Sweden	0.52***<br>(0.03)	0.56***<br>(0.08)	0.53***<br>(0.09)	0.50***<br>(0.09)	0.48***<br>(0.07)	0.53***<br>(0.06)
28	Switzerland	0.47***<br>(0.02)	0.41***<br>(0.05)	0.53***<br>(0.05)	0.49***<br>(0.05)	0.42***<br>(0.04)	0.50***<br>(0.05)
29	United Kingdom	0.47***<br>(0.03)	0.49***<br>(0.10)	0.49***<br>(0.07)	0.43***<br>(0.07)	0.50***<br>(0.08)	0.44***<br>(0.07)
30	United States	0.34***<br>(0.04)	0.44***<br>(0.08)	0.27***<br>(0.07)	0.38***<br>(0.07)	0.25*<br>(0.08)	0.40***<br>(0.08

Standard errors in parentheses.
* $p < 0.05$, ** $p < 0.01$, *** $p < 0.001$.

Table S7. Standardized sex differences (men–women) in problem-solving as intraindividual strengths by country and age groups.

		Sex differences in problem-solving as IIS					
			Age categories				
		Overall	24 or less	25-34	35-44	45-54	55 plus
Serial	Country	Coefficient (female)	Coefficient (female)	Coefficient (female)	Coefficient (female)	Coefficient (female)	Coefficient (female)
1	Austria	-0.01 (0.03)	0.02 (0.12)	0.12 (0.11)	-0.03 (0.10)	-0.04 (0.10)	-0.12 (0.09)
2	Belgium	-0.04 (0.03)	0.08 (0.06)	-0.06 (0.07)	-0.11 (0.06)	-0.07 (0.06)	-0.01 (0.05)
3	Canada	-0.21*** (0.04)	-0.34** (0.10)	-0.20 (0.08)	-0.27** (0.08)	-0.20* (0.07)	-0.11 (0.06)
4	Chile	0.09 (0.04)	0.24** (0.07)	0.19 (0.07)	-0.04 (0.08)	0.03 (0.08)	0.04 (0.07)
5	Croatia	0.08 (0.03)	0.11 (0.07)	0.08 (0.04)	0.18* (0.06)	0.04 (0.06)	0.04 (0.06)
6	Czech Republic	-0.02 (0.05)	-0.05 (0.03)	-0.05 (0.08)	0.02 (0.08)	-0.04 (0.06)	-0.01 (0.07)
7	Denmark	-0.03 (0.03)	-0.20 (0.08)	-0.04 (0.07)	0.05 (0.06)	0.01 (0.06)	-0.00 (0.05)
8	Estonia	0.04 (0.02)	0.02 (0.05)	0.09 (0.04)	-0.02 (0.04)	0.00 (0.04)	0.06 (0.04)
9	Finland	0.08 (0.03)	0.03 (0.07)	0.04 (0.07)	0.10 (0.06)	0.04 (0.06)	0.15 (0.05)
10	France	-0.04 (0.02)	-0.06 (0.05)	-0.10 (0.06)	-0.03 (0.05)	-0.03 (0.04)	-0.01 (0.05)
11	Germany	-0.04 (0.02)	-0.04 (0.05)	-0.04 (0.05)	-0.05 (0.05)	-0.06 (0.05)	-0.02 (0.05)
12	Hungary	0.03 (0.02)	-0.01 (0.06)	-0.00 (0.05)	0.07 (0.05)	-0.01 (0.04)	0.09 (0.05)
13	Ireland	-0.05 (0.03)	0.15 (0.10)	-0.07 (0.09)	-0.03 (0.06)	-0.21 (0.07)	-0.05 (0.06)
14	Israel	0.02 (0.03)	0.07 (0.06)	0.02 (0.06)	0.03 (0.06)	0.03 (0.06)	-0.05 (0.06)
15	Italy	0.04 (0.03)	-0.02 (0.06)	0.04 (0.08)	0.13 (0.08)	0.09 (0.07)	-0.04 (0.07)

16	Japan	-0.20*** (0.04)	-0.22* (0.06)	-0.18* (0.05)	-0.17 (0.05)	-0.22** (0.04)	-0.21* (0.05)
17	Korea	-0.05 (0.02)	0.15 (0.08)	0.12 (0.05)	0.05 (0.04)	0.08 (0.05)	-0.08 (0.04)
18	Latvia	-0.02 (0.03)	-0.15 (0.11)	-0.09 (0.08)	0.02 (0.07)	0.07 (0.06)	-0.05 (0.06)
19	Lithuania	0.14** (0.05)	-0.05 (0.08)	0.10 (0.06)	0.24** (0.06)	0.12 (0.06)	0.21* (0.05)
20	New Zealand	-0.04 (0.06)	0.03 (0.09)	0.16 (0.11)	-0.22* (0.09)	-0.22 (0.15)	0.00 (0.08)
21	Norway	-0.04 (0.05)	0.06 (0.07)	-0.05 (0.06)	-0.05 (0.06)	-0.13 (0.06)	-0.01 (0.05)
22	Poland	0.02 (0.05)	-0.03 (0.06)	0.11 (0.06)	0.03 (0.05)	-0.02 (0.05)	0.00 (0.06)
23	Portugal	0.09 (0.06)	0.10 (0.08)	0.15 (0.07)	0.22* (0.08)	0.01 (0.07)	-0.02 (0.08)
24	Singapore	-0.00 (0.04)	0.05 (0.06)	0.01 (0.05)	0.01 (0.05)	-0.01 (0.05)	-0.05 (0.05)
25	Slovak Republic	-0.04 (0.05)	-0.18 (0.08)	-0.03 (0.07)	-0.05 (0.06)	0.03 (0.06)	-0.01 (0.07)
26	Spain	-0.18*** (0.04)	-0.29* (0.07)	-0.34 (0.07)	-0.18* (0.05)	-0.16* (0.05)	-0.25** (0.05)
27	Sweden	-0.13** (0.05)	-0.17 (0.07)	-0.14 (0.08)	-0.17 (0.08)	-0.11 (0.07)	0.08 (0.06)
28	Switzerland	-0.14** (0.02)	-0.03 (0.05)	-0.17 (0.05)	-0.14 (0.05)	-0.14 (0.05)	-0.18* (0.05)
29	United Kingdom	-0.17** (0.05)	-0.12 (0.10)	-0.17 (0.08)	-0.13 (0.06)	-0.24* (0.08)	-0.17 (0.07)
30	United States	0.00 (0.05)	0.07 (0.08)	0.02 (0.07)	-0.04 (0.08)	0.02 (0.08)	-0.08 (0.07)

Standard errors in parentheses.
* $p < 0.05$, ** $p < 0.01$, *** $p < 0.001$.

Figure S1 Correlation between sex differences in literacy and numeracy as intraindividual strengths.

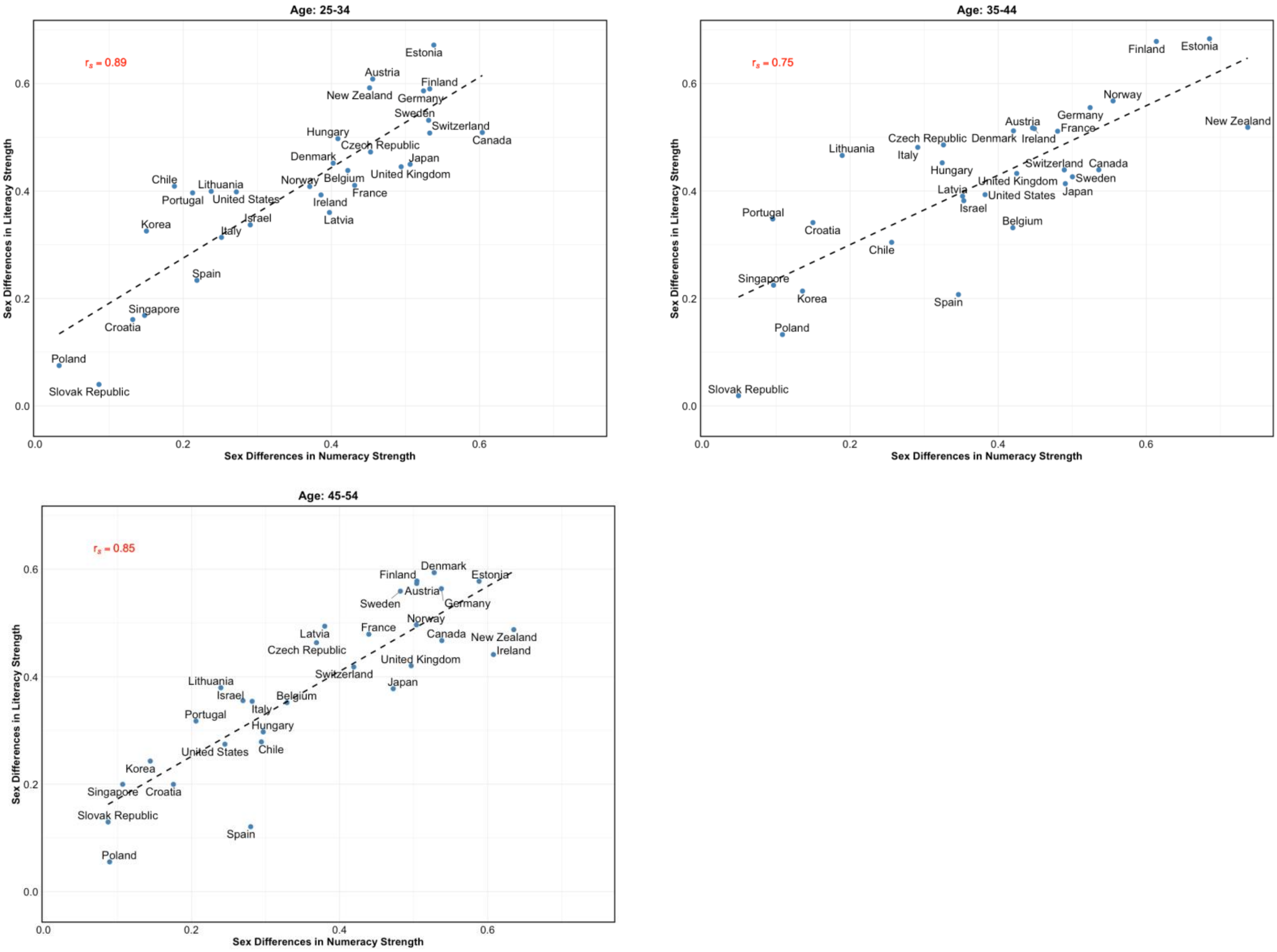

Spearman correlation (rs) between standardized sex differences in literacy (women > men) and numeracy (men > women) as intraindividual strengths across age groups. To aid interpretation, sex differences in literacy were multiplied by +1.
